# Therapeutic potential of nimotuzumab PEGylated-maytansine antibody drug conjugates against EGFR positive xenograft

**DOI:** 10.18632/oncotarget.26613

**Published:** 2019-02-01

**Authors:** Siddesh V. Hartimath, Ayman El-Sayed, Amal Makhlouf, Wendy Bernhard, Carolina Gonzalez, Wayne Hill, Angel Casaco Parada, Kris Barreto, Clarence Ronald Geyer, Humphrey Fonge

**Affiliations:** ^1^ Department of Medical Imaging, College of Medicine, University of Saskatchewan, Saskatoon SK, S7N 0W8, Canada; ^2^ Saskatchewan Centre for Cyclotron Sciences (SCCS), the Fedoruk Centre, Saskatoon SK, S7N 5C4, Canada; ^3^ Department of Pathology and Laboratory Medicine, College of Medicine, University of Saskatchewan, Saskatoon SK, S7N 5E5, Canada; ^4^ Department of Pharmaceutics and Industrial Pharmacy, Faculty of Pharmacy, Cairo University, Kasr El-Aini, 12411, Cairo, Egypt; ^5^ Centre for Molecular Immunology, Havana, 11600, Cuba; ^6^ Department of Medical Imaging, Royal University Hospital Saskatoon, Saskatoon SK, S7N 0W8, Canada

**Keywords:** epidermal growth factor receptor I, antibody drug conjugates, near infrared imaging, PEGylated maytansine, colorectal cancer

## Abstract

Nimotuzumab is a humanized anti-epidermal growth factor receptor I (EGFR) monoclonal antibody. We have developed antibody drug conjugates (ADCs) with nimotuzumab conjugated to PEGylated-maytansine (PEG_6_-DM1). We generated conjugates with low (nimotuzumab-PEG_6_-DM1-Low: DAR = 3.5) and high (nimotuzumab-PEG_6_-DM1-High: DAR = 7.3) drug to antibody ratios (DAR). Quality control was performed using UV spectrophotometry, size exclusion HPLC, bioanalyzer, biolayer interferometry (BLI), and flow cytometry in EGFR-positive DLD-1, MDA-MB-468 (high density EGFR), and HT-29 (very low EGFR density) cells. Control antibody drug conjugates were developed using a human anti-maltose binding protein (MBP) antibody. BLI showed that the binding of nimotuzumab-PEG_6_-DM1-Low and nimotuzumab-PEG_6_-DM1-High was slightly but significantly affected by conjugation of the drug (nimotuzumab K_D_ 0.89 ± 0.02 nM < nimotuzumab-PEG_6_-DM1-Low K_D_ 1.94 ± 0.02 nM < nimotuzumab-PEG_6_-DM1-High K_D_ 3.75 ± 0.03 nM). *In vitro* cytotoxicity was determined following incubation of cells with the immunoconjugates and IC_50_ values were determined. Nimotuzumab-PEG_6_-DM1-Low and nimotuzumab-PEG_6_-DM1-High were used to treat EGFR positive KRAS mutant DLD-1 colorectal cancer xenograft. DLD-1 cells were transduced with a red fluorescent protein (iRFP702) to allow the use of near infrared imaging (NIR) for tumor response monitoring. *In vitro* potency correlated with the number of drugs on antibody, with nimotuzumab-PEG_6_-DM1-High showing higher activity than nimotuzumab-PEG_6_-DM1-Low. Three doses (15 mg/kg) of the ADCs prolonged the survival of DLD-1-iRFP-702 tumor bearing mice as monitored by NIR. Nimotuzumab-PEG_6_-DM1-Low resulted in 4/6 complete cure while nimotuzumab-PEG_6_-DM1-High resulted in 2/5 complete cure. The novel ADCs were very effective in a colorectal cancer model *in vivo*.

## INTRODUCTION

The human epidermal growth factor receptor (EGFR), also known as EGFR/ErbB 1/HER 1 is a 170 kDa transmembrane cell surface glycoprotein which belongs to the subfamily of type-1 tyrosine kinase receptor. Other members of this family include, ErbB-2/HER-2, ErbB-3/HER-3 and ErbB-4/HER-4 [1]. Overexpression of EGFR is implicated in all aggressive cancers of epithelial origin including squamous cell head & neck (90–100%) [2], glioma (90–100%) [3], non-small cell lung (75–90%), colorectal (80–85%) [4], breast (20–30%) [5] and cervical [6] cancers. Mutations in EGFR lead to worse or unpredictable outcomes in patients treated with anti-EGFR drugs. This is particularly the case with kirsten rat sarcoma viral oncogene (KRAS), an intracellular effector molecule that routs ligand-bound EGFR to the nucleus, where it leads to constitutive phosphorylation of the receptor and hence tumor proliferation.

Anti-EGFR antibodies e.g. cetuximab [7], panitumumab [8] and nimotuzumab [9, 10] are used to treat different EGFR positive cancers. With the exception of nimotuzumab, these antibodies are associated with significant cutaneous toxicity in 45–100% of patients [11–13]. In contrast, nimotuzumab is better tolerated [10, 14] and has low skin toxicity, because its “affinity optimized” binding characteristic ensures low transient binding to low EGFR-expressing healthy tissues such as the skin. Garrido *et al.* [15] showed that the low skin toxicity of nimotuzumab is attributed to its transient monovalent binding in low-EGFR expressing tissues such as the skin and gastro-intestinal mucosa (these tissues account for the dose-limiting toxicities observed with other anti-EGFR antibody treatments). This low transient monovalent binding is due to a 10-fold lower affinity of nimotuzumab for EGFR compared with other anti-EGFR antibodies such as cetuximab or panitumumab [15]. Due to its higher binding affinity, bivalent stable binding is observed with cetuximab even in low EGFR expressing tissues [15].

The efficacy of anti-EGFR antibodies is poor [16, 17]. The efficacy of these antibodies is improved by conjugation of multiple drugs to the antibody. Antibody drug conjugates (ADCs) are associated with improvements in efficacy compared with (unconjugated) antibodies [18]. ABT-414 and AMG-595 are two anti-EGFR ADCs in phase I/II trials. In AMG-595, the antibody is conjugated to a cytotoxic agent maytasine (DM1) [19]. Anti-Her2 monoclonal antibody trastuzumab conjugated to DM1 (Kadcyla^®^, trastuzumab emtansine) is effective in preclinical models and in patients with Her2-positive trastuzumab- or lapatinib-resistant phenotypes and is currently approved [20, 21]. Despite some improvements in efficacy with ADCs versus antibodies, acquired resistance is common. Acquired resistance to chemotherapy and ADCs is often due to the expression of multidrug resistant gene (MDR1), a drug efflux pump. Many cytotoxic small molecules including DM1 are substrates for MDR1 and are actively pumped out of the cell [22–24]. Recently, it has been shown that ADCs developed with PEGylated DM1 (PEG-DM1) are more potent *in vitro* than those with DM1 [24, 25]. These PEGylated ADCs are also more hydrophilic making it possible to conjugate many drugs to the antibodies without adverse effects on the binding to antigens [24, 25].

Metastatic colorectal cancer (mCRC) is the second leading cause of death from cancer with a 5-year survival rate of <10% (stage IV) [26], likely due to the fact that over 45% of CRC patients have metastatic disease at initial diagnosis. Surgery, which is a primary treatment option is contraindicated in patients with advanced disease and even when this is possible, local recurrence rates after surgery is very high (38–88%) [27]. About 80% of CRC patients overexpress EGFR [28, 29]. Mutations in the KRAS oncogene (present in 40% of mCRC) lead to constitutive over-activation of EGFR and drive *de novo* resistance to anti-EGFR drugs [30–32]. In EGFR-positive mCRC patients with wild-type KRAS, the addition of anti-EGFR antibodies (e.g. cetuximab and panitumumab) to chemotherapy results in significant, albeit small improvements in survival; there is no observed benefit in patients with KRAS mutations [17, 33–35]. Here, we synthesized nimotuzumab drug conjugates with low (nimotuzumab-PEG_6_-DM1-Low: 3–4 drugs/antibody) and high (nimotuzumab-PEG_6_-DM1-High: 7–8 drugs per antibody) drug to antibody (DAR) ratios. The nimotuzumab drug conjugates were evaluated *in vitro* and *in vivo* for their efficacy against EGFR-positive cancer cells and KRAS mutant mCRC mouse xenograft.

## RESULTS

### Synthesis of drug-linker and antibody conjugation

In order to develop stable ADCs, it is important to make the cytotoxic drug with a suitable linker. The commonly used linkers such as N-succinimidyl-4-(2-pyridyldithio) butanoate (SPDB) and succinimidyl 4-(N-maleimidomethyl)cyclohexane-1-carboxylate (SMCC) give rise to a DARs of 4–5. Previous attempts to make ADCs with higher DARs resulted in the formation of aggregates and abrogation of antigen binding. Hence, we used the bifunctional a poly-ethylene glycol (PEG_6_) linker with maleimide and N-hydroxysuccinamide (NHS) functional groups (Mal-PEG_6_-DM1-NHS). The maleimide offers a stable non-cleavable thioether linker with DM1, whereas NHS reacts with primary amines of the antibody. Supplementary Scheme 1 (Supplementary Materials) shows the synthetic scheme for drug linker and ADC synthesis. Conjugation of the drug with the bifunctional linker was confirmed using NMR and mass spectrometry (MS). A yield of 67.4% was obtained and the drug linker was used to conjugate the antibodies without further purification. The antibody conjugation reactions were optimized in different buffers such as PBS, sodium carbonate and HEPES at different pH values in order to obtain the desired DAR. We aimed for an average DAR of 3–4 for nimotuzumab-PEG_6_-DM1-Low and 7–8 for nimotuzumab-PEG_6_-DM1-High. To avoid precipitation the nimotuzumab-PEG_6_-DM1-High reactions were carried out with <1% DMSO for a longer reaction time of up to 20 h. Under optimal reaction conditions (0.1 M HEPES, pH 8.5, incubation time 2 h at 37° C and further incubation at 4° C for 20 h) the desired DAR was obtained. For nimotuzumab-PEG_6_-DM1-Low an eight-fold excess of the linker to the antibody was used, while for nimotuzumab-PEG_6_-DM1-High, a 16-fold excess of the linker to the antibody was used. The DAR was determined using UV spectroscopy and was 3.5 ± 0.2 (*n* = 15) for nimotuzumab-PEG_6_-DM1-Low and 7.3 ± 0.4 (*n* = 15) for nimotuzumab-PEG_6_-DM1-High (Supplementary Figure 1). The spectra of conjugated and unconjugated IgG were sufficiently different to detect the absorption maxima of the drug and the antibody. In addition, it was also evident that nimotuzumab-PEG_6_-DM1-Low and nimotuzumab-PEG_6_-DM1-High showed a distinct difference in UV absorbance at 254 nm.

### Characterization of ADCs

Quality control of ADCs were done using SEC-HPLC with PBS (pH 7.2) as the mobile phase (Supplementary Figure 2). Antibody eluted as a monomer peak at 12.9 min. Antibody aggregates and dimers, if present eluted at 8.7 min and 10.8 min, respectively. Aggregate or dimer formation for nimotuzumab-PEG_6_-DM1-Low or nimotuzumab-PEG_6_-DM1-High were <3% and the presence of the free drug was not detected. Free drug PEG_6_-DM1 elutes at 24.2 min and its degraded products later (data not shown).

**Figure 1:**
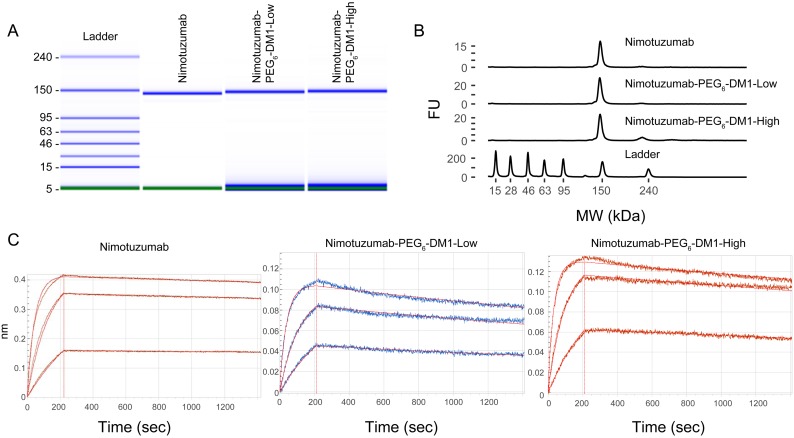
(**A**–**C**) Quality control of ADCs using bioanalyzer (**A**, **B**) and biolayer interferometry (BLI) (**C**). A, B: Bioanalyzer shows electropherogram of the ladder (L), nimotuzumab (1), nimotuzumab-PEG_6_-DM1-Low (2) and nimotuzumab-PEG_6_-DM1-High (3). BLI binding curves for nimotuzumab, nimotuzumab-PEG_6_-DM1-Low, nimotuzumab-PEG_6_-DM1-High at different concentrations (55, 166 and 500 nM).

Electronic electrophoresis (bioanalyzer) was used to determine molecular weights and purities of the ADCs. The molecular weight of nimotuzumab was 140.1 kDa, while nimotuzumab-PEG_6_-DM1-Low and nimotuzumab-PEG_6_-DM1-High was 144.6 and 149.8 kDa, respectively. The formation of aggregates was <1% in both the conjugates (Figure 1A and 1B). Based on the molecular weights determined from the bioanalyzer, the average DAR was found to be 3.3 and 7.2 for nimotuzumab-PEG_6_-DM1-Low and nimotuzumab-PEG_6_-DM1-High, respectively.

### Binding kinetics

Binding kinetics were measured using biolayer interferometry performed using immobilized anti-human FAB-CH1 sensors. Binding kinetics were calculated using a 1 to 1 fitting model, and a dissociation constant (K_D_) was obtained. Both nimotuzumab and nimotuzumab ADCs showed similar association rate to EGFR. Whereas, there was a significant decrease in the dissociation rate in both nimotuzumab-PEG_6_-DM1-Low (*p* = 0.017) and nimotuzumab-PEG_6_-DM1-High (*p* = 0.0009) compared with nimotuzumab (Table 1). K_D_ values for nimotuzumab, nimotuzumab-PEG_6_-DM1-Low and nimotuzumab-PEG_6_-DM1-High were, 0.89 ± 0.02 nM, 1.94 ± 0.02 nM, and 3.75 ± 0.03 nM, respectively. Both the nimotuzumab-PEG_6_-DM1-Low and nimotuzumab-PEG_6_-DM1-High conjugates showed ~ 2.2-fold and 4.2-fold increase in K_D_, respectively, compared to nimotuzumab (Figure 1C).

**Table 1:** Binding constants of antibodies and antibody drug conjugates

Sample	K_D_ (M)	K_D_ Error	k_on_(1/Ms)	k_on_ Error	k_dis_(1/s)	k_dis_ Error
Nimotuzumab	8.99 × 10^−10^	1.61 × 10^−11^	4.43 × 10^4^	1.68 × 10^2^	3.98 ×10^5^	6.98 × 10^−7^
Nimotuzumab-PEG_6_-DM1-High	3.75 × 10^−9^	2.84 × 10^−11^	5.15 × 10^4^	2.74 × 10^2^	1.93 × 10^4^	1.04 ×10^−6^
Nimotuzumab-PEG_6_-DM1-Low	1.94 × 10^−9^	1.59 × 10^−11^	6.10 ×10^4^	2.69 × 10^2^	1.19 ×10^4^	8.18 × 10^−7^

### Flow cytometry

**Figure 2:**
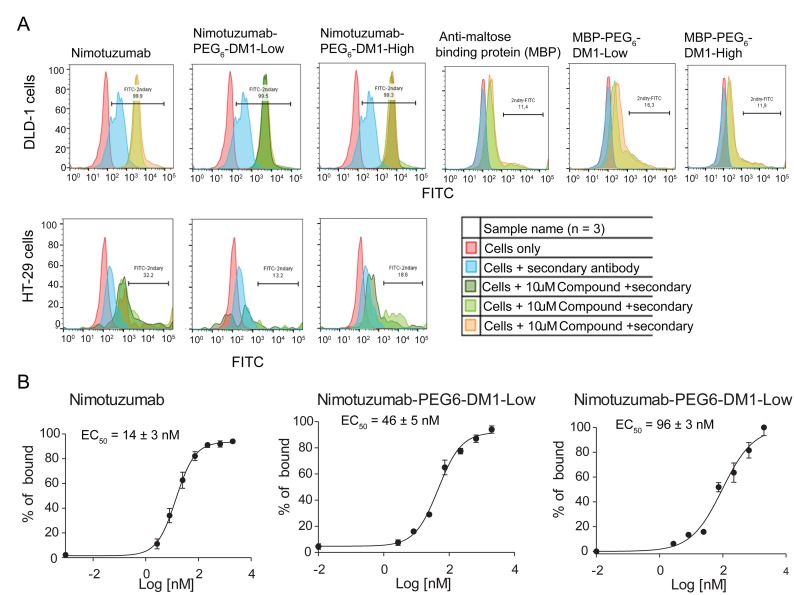
(**A**, **B**) *In-vitro* flow cytometry binding assay. (**A**) Cells were incubated with nimotuzumab, nimotuzumab-PEG_6_-DM1-Low and nimotuzumab-PEG_6_-DM1-High at 10 μM. Binding of antibody and ADCs to EGFR on cells was estimated by adding a secondary antibody labelled with FITC. Untreated cells and cells with only secondary antibody were used as controls. All the samples were run in triplicate. (**B**) 8-point curve flow cytometry experiments were performed in order to estimate the potency of nimotuzumab and nimotuzumab ADCs in DLD-1 cells. Percentage of bound was estimated from the relative fluorescence intensity and plotted against concentration. A non-linear curve fitting was used to estimate the EC50 of nimotuzumab, nimotuzumab-PEG_6_-DM1-Low and nimotuzumab-PEG_6_-DM1-High were 14 ± 3 nM, 46 ± 5 nM, and 96 ± 3 nM, respectively.

To determine if the conjugation of drug linker altered the binding of ADCs to EGFR- positive cells, we performed *in vitro* binding using flow cytometry. One-point binding of nimotuzumab, nimotuzumab-PEG_6_-DM1-Low nimotuzumab-PEG_6_-DM1-High and control ADCs was measured against in EGFR positive DLD-1, MDA-MB-468 (high EGFR expression) and HT-29 (low EGFR expression) cell lines (Figure 2A). Based on the relative fluorescence intensity, there was no change in the binding affinity of nimotuzumab-PEG_6_-DM1-Low and nimotuzumab-PEG_6_-DM1-High conjugates compared to nimotuzumab at concentration of 10 μM. To show that the binding of nimotuzumab was specific, control anti-MBP IgG was used and subjected to the same conjugations as nimotuzumab. Flow data showed that there was a significant reduction in binding of anti-MPB IgG and anti-MBP ADCs in EGFR-positive (DLD-1) (Figure 2A). This indicates that nimotuzumab-PEG_6_-DM1-Low and nimotuzumab-PEG_6_-DM1-High binding was specific. To determine the binding constant to DLD-1 cells an 8-point saturation curve by flow cytometry was done. Median Fluorescence Intensity (MFI) was converted into percent bound and plotted against concentration to calculate K_D_ values for DLD-1 cells. The estimated K_D_ values for nimotuzumab, nimotuzumab-PEG_6_-DM1-Low and nimotuzumab-PEG_6_-DM1-High were 14 ± 3 nM, 46 ± 5 nM and 96 ± 3 nM, respectively (Figure 2B). There was a 4- and 7-fold increase in K_D_ values between nimotuzumab-PEG_6_-DM1-Low and nimotuzumab-PEG_6_-DM1-High, respectively, compared to nimotuzumab. We observed a significant difference in binding between nimotuzumab and nimotuzumab-PEG_6_-DM1-High (14 ± 3 nM vs 96 ± 3 nM; *p* < 0.05). Whereas, there was no statistical difference between nimotuzumab and nimotuzumab-PEG_6_-DM1-Low (14 ± 3 nM vs 46 ± 5 nM; *p* = 0.09).

### *In vitro* cytotoxicity

**Figure 3:**
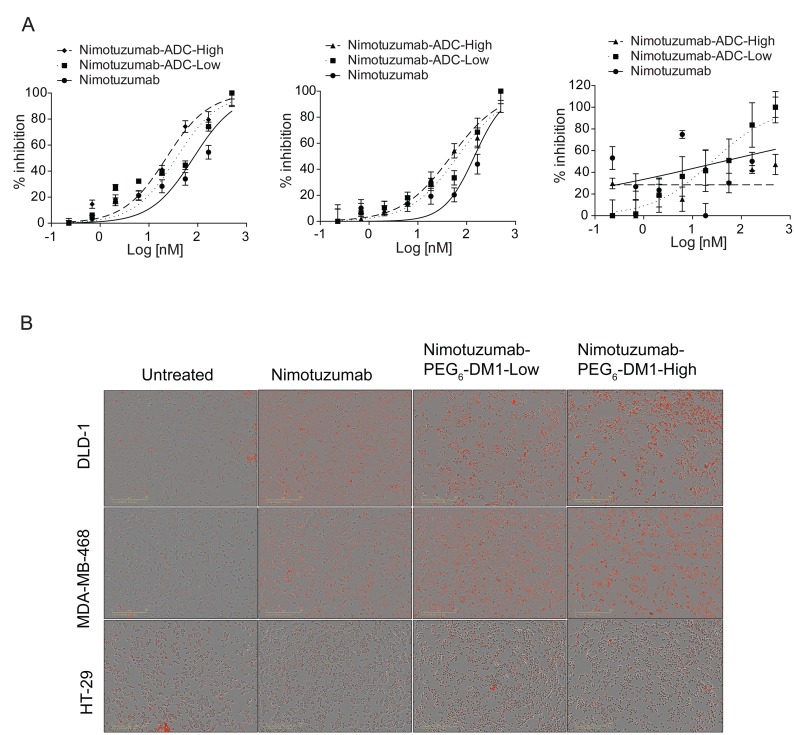
(**A**) *In vitro* cytotoxic assay against DLD-1, MDA-MD468 and HT-29 cells. The potency of nimotuzumab and nimotuzumab-ADCs were estimated by plotting the increased cytotoxic red intensity as a function of cell death against the concentration. A non-linear curve fitting was used to estimate the potency, and all the experiments were run in triplicate. (**B**) Representative phase contrast images of cells treated with nimotuzumab and nimotuzumab-PEG_6_-DM1-Low and nimotuzumab-PEG_6_-DM1-High from cytotoxic assay.

Live cell imaging experiments were performed to determine the cytotoxicity of ADCs in different cell lines. After adding nimotuzumab, nimotuzumab-PEG_6_-DM1-Low and nimotuzumab-PEG_6_-DM1-High, cells were monitored for 72 h without changing the media. Nimotuzumab-PEG_6_-DM1-High which has a higher DAR was more cytotoxic to EGFR positive cells (Figure 3A). The IC_50_ of nimotuzumab, nimotuzumab-PEG_6_-DM1-Low and nimotuzumab-PEG_6_-DM1-High was 80.8 ± 5 nM, 36.2 ± 2 nM, and 22.5 ± 1 nM, respectively against DLD-1 cells. The IC_50_ value of nimotuzumab-PEG_6_-DM1-High in DLD-1 was significantly lower when compared with nimotuzumab (22.5 ± 1 nM vs 80.8 ± 5 nM; *p* < 0.01). Similarly, the IC_50_ of nimotuzumab-PEG_6_-DM1-Low was lower than for nimotuzumab (36.2 ± 2 nM vs 80.8 ± 5 nM; *p* < 0.05). The IC_50_ of nimotuzumab-PEG_6_-DM1-High was significantly lower than nimotuzumab-PEG_6_-DM1-Low (22.5 ± 1 nM vs 36.2 ± 2 nM; *p* < 0.05). A similar effect was seen against MDA-MB-468 cell line with IC_50_ values 144.3 ± 3 nM, 65.5 ± 6 nM and 48.1 ± 8 nM for nimotuzumab, nimotuzumab-PEG_6_-DM1-Low and nimotuzumab-PEG_6_-DM1-High, respectively. There was no true fit of the data for nimotuzumab and the ADCs in HT-29 (*R*^2^ = 0.16, *n* = 3, Figure 3A) indicating that there was little receptor mediated cell killing for this low EGFR expressing cell line. Furthermore, phase contrast images revealed that at higher concentration (500 nM, red fluorescence), there was significant cell death within 10 h after treatment in DLD-1 and MDA-MB-468 cell lines when compared with untreated cells (Figure 3B). However, there was less cell death in HT-29 when compared with treated and untreated wells.

### Normal tissue toxicity of antibody drug conjugates

Single dose (15 mg/kg, 25 mg/kg or 50 mg/kg) delayed (Day-14) toxicity was studied in normal Balb-C mice after administration of PBS, nimotuzumab, nimotuzumab-PEG_6_-DM1-Low or nimotuzumab-PEG_6_-DM1-High. There was a dose-dependent toxicity in mice treated with ADCs. There was no difference in any CBC parameters in mice administered with 15 mg/kg nimotuzumab-PEG_6_-DM1-Low (Supplementary Table 1A) or nimotuzumab-PEG_6_-DM1-High (Supplementary Table 1B) compared with PBS treated mice. However, significant changes were seen in a few CBC parameters in mice treated with 25 mg/kg and 50 mg/kg doses (Supplementary Table 1A and 1B). There were no differences in the clinical chemistry between PBS injected mice and 15 mg/kg doses of nimotuzumab-PEG_6_-DM1-Low (Supplementary Table 2A) or nimotuzumab-PEG_6_-DM1-High (Supplementary Table 2B). Significant deviations in some values were seen at higher doses (25 mg/kg and 50 mg/kg). (Supplementary Table 2A and 2B). No deaths occurred in any treatment group and there were no significant differences in body weight (data not shown) between all the groups. There was no significant loss in body weight in all treatment groups (data not shown) during the study period.

### Efficacy of antibody drug conjugates in DLD-1-IRFP-702 xenograft

**Figure 4:**
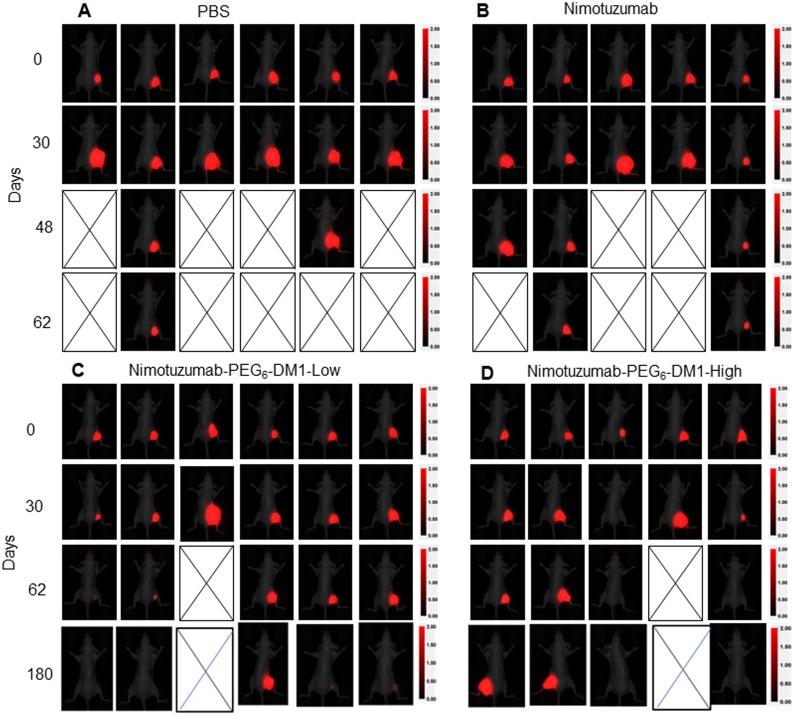
Whole body near infrared imaging of the EGFR-positive murine xenograft model DLD-1-iRFP-702 following treatment with (**A**) Normal saline, (**B**) nimotuzumab (**C**) nimotuzumab-PEG_6_-DM1-Low, and (**D**) nimotuzumab-PEG_6_-DM1-High. Mice were treated with three doses (15 mg/kg) of nimotuzumab or nimotuzumab ADCs or with an equivalent volume of normal saline on day 0, 8 and 16. Representative near-infrared posterior whole-body images merged with white light images of CD-1 nude mice bearing subcutaneous DLD-1-iRFP-702 xenografts (right hind flank) are presented.

We evaluated the therapeutic efficacy of nimotuzumab-PEG_6_-DM1-Low and nimotuzumab-PEG_6_-DM1-ADC-high in EGFR-positive DLD-1-IRFP-702 xenograft. A 15 mg/kg dose of nimotuzumab, nimotuzumab-PEG_6_-DM1-Low or nimotuzumab-PEG_6_-DM1-ADC-high was intravenously administered to DLD-1-IRFP-702 tumor bearing mice. Efficacy was evaluated by measuring change in tumor volume using caliper measurements or using near infrared imaging. NIR images of mice were obtained for up to day-180 (Figure 4A–4C). In PBS treated groups, one mouse reached the study end point (2000 mm^3^) by day-62 and the other 5 had to be sacrificed prior to day-62 (Figure 4A). In the nimotuzumab group two mice were alive at day-62 (Figure 4B). The two mice showed some response to nimotuzumab therapy evidenced by slowed xenograft growth. In the nimotuzumab-PEG_6_-DM1-Low group four out of six mice showed complete cure by day-180, while one mouse showed moderate response to therapy evidenced by slow growth of xenograft (Figure 4C). Of the four mice with complete cure, two of them were cured at day-62 and tumors did not regrow (>180 days after treatment). One mouse out of 6 did not respond to therapy and had to be sacrificed on day-30. In the nimotuzumab-PEG_6_-DM1-High two out of 5 mice had complete cure by day-62, while two other mice showed slowed growth of xenografts (Figure 4D). Similar to the nimotuzumab-PEG_6_-DM1-Low group, one mouse in nimotuzumab-PEG_6_-DM1-High group did not respond to therapy and had to be sacrificed on day-30.

**Figure 5:**
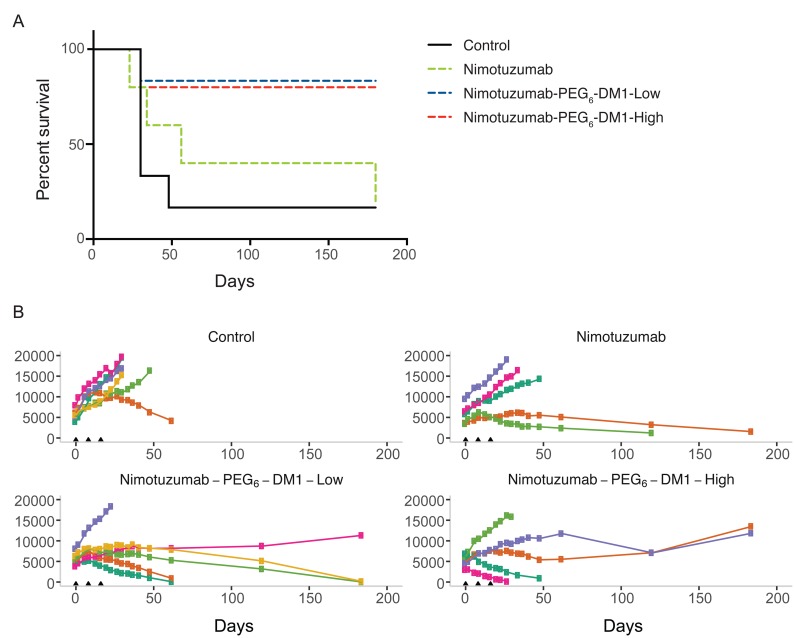
(**A**–**B**) Enhanced efficacy of nimotuzumab ADCs in EGFR-positive murine xenograft model DLD-1-iRFP-702 (A) Kaplan–Meier curves for overall survival in CD-1 mice bearing DLD-1-iRFP-702 xenografts following treatment with normal PBS (black solid line), nimotuzumab (green dotted line), nimotuzumab-PEG_6_-DM1-Low (blue dotted line), and nimotuzumab-PEG_6_-DM1-High (red dotted line). When xenografts reached the appropriate size, mice were randomized into 5–6 mice per group and treated on day 0, 8 and 16. The endpoint was when xenograft volume reached 2000 mm^3^, or if the xenograft ulcerated (> 20% of tumor volume). Log-rank test analysis demonstrated that the survival of the PBS group was significantly different from that of nimotuzumab-PEG_6_-DM1-Low group and nimotuzumab-PEG_6_-DM1-High group (*p* < 0.05). While there was no statistically significant difference between normal saline group and nimotuzumab group survival curves (*p* > 0.05). (B) Tumor volumes of individual mice (represented by the different colors) as measured (see Supplementary Materials for details) by whole body near infrared imaging of (fluorescently labelled) DLD-1-iRFP-702 tumor bearing mice.

Kaplan Meier survival curves for the different groups were calculated (Figure 5A). The survival of the PBS group was significantly lower than nimotuzumab-PEG_6_-DM1-Low and nimotuzumab-PEG_6_-DM1-High group (*p* < 0.05, Log-rank test). There was no statistically significant difference between the PBS and nimotuzumab group's survival curves (*p* > 0.05, Log-rank test). The median survival of the PBS group was 30 days, while that of nimotuzumab group was 56 days. On the other hand, median survival of the nimotuzumab-PEG_6_-DM1-Low and nimotuzumab-PEG_6_-DM1-High groups had not been reached (>180 days after the start of treatment). None of the treatment groups resulted in weight loss as all mice gained weight at the same rate (Supplementary Figure 3). NIR images were extracted and analysed, and used to generate tumor growth curves (Figure 5B). There was a statistically significant difference in the xenograft growth index between therapy groups vs. the control saline group (*p* < 0.05, Two-way ANOVA test followed by Tukey's multiple comparison test). Caliper measurements of tumor volumes (Supplementary Figure 4) showed variability in volume measurements compared with NIR image-analysed volumes (Figure 5B)

## DISCUSSION

Several factors account for the reason why antibodies have not lived up to their expectations as immunotherapeutics. *De novo* and acquired resistance to immunotherapeutics is a common phenomenon in oncology. Cancer cells develop intrinsic resistance (acquired resistance) by frequently overexpressing alternate growth factors and their receptors. EGFR overexpression for example is a widely accepted mechanism for acquired resistance to epidermal growth factor receptor II (Her2) targeted treatments [36]. Overexpression of MDR1 is a widely accepted mechanism of *de novo* resistance by cancer cells to small molecule drugs [22–24]. DM1 has been one of the drugs of choice of many ADCs. Once internalized the DM1 is cleaved from the antibody in lysosomes and becomes toxic to the cancer cell. PEGylated DM1 (PEG-DM1), as proposed in this study, was recently shown to be more potent than DM1 [24, 25]. An optimum DAR is needed to preserve biological characteristics of the antibody. Zhao *et al.* showed that a DAR of 9 using PEG-DM1 yielded ADCs that were more cytotoxic *in vitro* than the routinely used DAR of 3–4 [25]. In this study we developed nimotuzumab-PEG_6_-DM1-Low and nimotuzumab-PEG_6_-DM1-High ADCs with average DARs of 3.5 and 7.3, respectively. The DAR was confirmed by UV-spectrophotometry and electronic electrophoresis (bioanalyzer). Size exclusion HPLC and bioanalyzer showed that these ADCs were exclusively monomeric species with <3% of dimer/aggregates. ADCs with high DARs prepared using DM1 with sulfo-SMCC as bifunctional drug conjugation frequently results in highly aggregated antibodies with poor solubility and low immunoreactivity.

A critical factor that affects the binding of an ADC to the antigen is the exposure of chemical conditions to the antibody during conjugation and the number of drugs on the antibody. As such it is important to understand how this impacts the binding of the ADCs. In comparison with nimotuzumab, the binding of nimotuzumab-PEG_6_-DM1-Low and nimotuzumab-PEG_6_-DM1-High to recombinant EGFR was 2.2- and 4.2-fold lower, respectively. Interestingly, the association rates of nimotuzumab, nimotuzumab-PEG_6_-DM1-Low and nimotuzumab-PEG_6_-DM1-High were not significantly different (Table 1). However, dissociation rates were significantly different for nimotuzumab vs nimotuzumab-PEG_6_-DM1-High (*p* = 0.0009) and even more different for nimotuzumab vs nimotuzumab-PEG_6_-DM1-Low (*p* = 0.0171), indicating dissociation from the EGFR was in the order (slowest) nimotuzumab < nimotuzumab-PEG_6_-DM1-Low < nimotuzumab-PEG_6_-DM1-High. The fast dissociation of antibody-antigen complex in the case of nimotuzumab-PEG_6_-DM1-High resulted in poor binding to recombinant EGFR. Similarly, we have observed a similar trend in binding to EGFR positive DLD-1 cells. The K_D_ increased with an increasing number of DAR even though statistical significance was only seen when comparing nimotuzumab vs nimotuzumab-PEG_6_-DM1-High. The study by Zhao *et al*. did not analyse binding to recombinant EGFR but observed a slightly lower binding by flow cytometry when the unconjugated antibody was compared with antibody conjugates with a DAR of 6.8 but not when the DAR was between 3.3–3.9.

Live-cell imaging was used to study the *in vitro* cytotoxicity of the antibody and immunoconjugates. As expected the nimotuzumab-PEG_6_-DM1-High showed the highest toxicity to DLD-1, MDA-MB-468 and HT-29 cells. Despite its higher dissociation constant in these cells nimotuzumab-PEG_6_-DM1-High with 7–8 PEG_6_-DM1 drugs per antibody was the most potent compared with nimotuzumab-PEG_6_-DM1-Low or nimotuzumab. The IC_50_ in both cell lines was dependent on the DAR with nimotuzumab-PEG_6_-DM1-High > nimotuzumab-PEG_6_-DM1-Low > nimotuzumab in all cell lines tested (Figure 3). The potency of ADCs is dependent on lysosomal processing and release of cleaved lysine-SMCC-DM1 which is then toxic to cancer cells [37]. Kovtun *et al.* showed that the cytotoxicity of PEG_4_-Mal-DM1 conjugated to an anti-EpCAM antibody followed the same lysosomal proteolytic pathway (24). Anti-EpCAM-PEG_4_-DM1 which is processed by EpCAM positive cells resulting in a polar lysine-PEG_4_-DM1 metabolite which is cytotoxic to cells [24]. It is expected that nimotuzumab-PEG_6_-DM1 will be processed by EGFR positive cells similar to anti-EpCAM-PEG_4_-Mal-DM1 ADCs. Zhao *et al*. showed that ADCs with higher DAR were more potent than those with low DAR. In a separate study, we have developed DOTA conjugated nimotuzumab, nimotuzumab-PEG_6_-DM1-Low and nimotuzumab-PEG_6_-DM1-High, and radiolabeled these immunoconjugates with ^111^In (^111^In-nimotuzumab, ^111^In-nimotuzumab-PEG_6_-DM1-Low and ^111^In-nimotuzumab-PEG_6_-DM1-High) for microSPECT imaging and biodistribution studies (unpublished data). We showed that the rate of internalization was in the following order: ^111^In-nimotuzumab-PEG_6_-DM1-High > ^111^In-nimotuzumab-PEG_6_-DM1-Low > ^111^In-nimotuzumab. Since ADCs require lysosomal processing to be cytotoxic, this order of increasing internalization with increasing number of PEG_6_-DM1 further explains the observed trend in cytotoxicity. The ADCs caused minimal cytotoxicity to HT-29 cells with very low EGFR expression.

We studied the potential of nimotuzumab-PEG_6_-DM1-Low and nimotuzumab-PEG_6_-DM1-High to eradicate tumors *in vivo* using DLD-1 xenograft, a KRAS mutant (G13D) colorectal cancer model. Cetuximab or irinotecan used alone or in combination have poor efficacy in this model [38]. Toxicity studies showed that a single dose of up to 50 mg/kg of nimotuzumab-PEG_6_-DM1-Low or nimotuzumab-PEG_6_-DM1-High was well tolerated. However, a dose of 15 mg/kg was selected for this study. Mice received three 15 mg/kg doses of the antibody or immunoconjugates on days 0, 8 and 16. DLD-1 cells expressing NIR protein iRFP-702 allowed NIR imaging of the DLD-1-iRFP-702 xenograft. Tumor measurement using calipers suffers from intra and inter individual variability. We developed a protocol to measure tumor volume using NIR images on DLD-1-iRFP-702 xenograft. The advantages of this method include (1) reduction in variability of volume measurement, (2) reduction of bias introduced from unblended study design, and (3) improvement of inter-lab reproducibility of efficacy studies. We observed 4/6 complete cure in the nimotuzumab-PEG_6_-DM1-Low group compared with 2/5 in the nimotuzumab-PEG_6_-DM1-High group. This shows that the conjugate with the low DAR was more effective than the high DAR. This data contradicts the *in vitro* data which showed a lower IC_50_ for the nimotuzumab-PEG_6_-DM1-High than for the nimotuzumab-PEG_6_-DM1-Low DLD-1 treated cells. The slightly lower efficacy of nimotuzumab-PEG_6_-DM1-High compared to nimotuzumab-PEG_6_-DM1-Low can be explained by the lower tumor uptake of the former. Using single photon emission computed tomography (microSPECT) imaging studies in mice bearing DLD-1 tumor, we showed that tumor uptake of ^111^In- nimotuzumab-PEG_6_-DM1-High was significantly lower than for ^111^In-nimotuzumab-PEG_6_-DM1-Low and ^111^In-nimotuzumab at all imaging time points (unpublished data). These results explain why there was a slight decrease in efficacy of ^111^In-nimotuzumab-PEG_6_-DM1-High in DLD-iRFP-702 tumor bearing mice despite having a higher DAR.

We developed for the first time nimotuzumab antibody drug conjugates using a PEG_6_-DM1 drug linker. The PEGylated linker resulted in highly soluble ADCs with exclusively monomeric antibody molecules. Our nimotuzumab-PEG_6_-DM1-Low and nimotuzumab-PEG_6_-DM1-High conjugates were effective at eradicating DLD-1 xenograft *in vivo*. However, there was no observed advantage of the high drug ratio nimotuzumab-PEG_6_-DM1-High *in vivo* likely due to a combination of decreased affinity for EGFR and lower tumor uptake *in vivo*. Complete tumor cure without recurrence was observed for over four months using the immunoconjugates.

## MATERIALS AND METHODS

### General

All reagents and solvents were obtained from commercial suppliers and used without further purification. DM1 drug was obtained from Toronto Research chemicals (Toronto, ON, Canada) and NHS-PEG_6_-maleimide was purchased from Biochempeg (Watertown, MA, USA). DLD-1, MDA-MB-468 and HT-29 were obtained from ATCC (Manassas, VA, USA) and cultured in monolayers in RPMI-1640, DMEM and Myco's 5A media, respectively. All media was supplemented with 10% fetal calf serum and cells were maintained in a humidified atmosphere with 5% CO_2_ at 37° C. Proton magnetic resonance (^1^HNMR) spectra were obtained on a Bruker NMR and the exact mass was determined with TOF-MS (Waters).

### Synthesis and characterization of drug linker

Synthesis of maytansine conjugated to PEG_6_-NHS ester (DM1-PEG_6_-NHS) was achieved using a bifunctional linker Mal-PEG_6_-NHS and DM1 described previously [25]. Briefly, DM1 (73.8. 0.1 mmol) was dissolved in 1 mL of THF and 2-fold excess N-hydroxysuccinimide-polyethylene-glycol-6-maleimide (NHS-PEG_6_-Mal, 120.2 mg, 0.2 mmol) in 1.5 mL of THF:PBS (50 mM, pH 6) 2:1 v/v was added to the DM1 solution. The reaction was stirred at room temperature for 6 h and was monitored using thin layer chromatography (TLC). At the end of the reaction, the crude mixture was purified using column chromatography with silica gel eluting with ethanol/dichloromethane 6/94 v/v. The excess solvents and moistures were removed using ultra-high vacuum giving 80.1 mg (67.4%) of DM1-PEG_6_-NHS. H^1^ NMR (CDCl_3_, reference 7.26 ppm) δ 0.79 (6H,s), 1.21–1.34 (16H,m), 1.41–1.52 (3H, s), 1.62–1.73 (6H,s), 2.01–2.21 (1H,d), 2.22–2.64 (4H,m), 2.71–2.75 (4H,s), 2.82–3.01(10H,m), 3.02–3.20 (5H,s), 3.31–3.42(5H,m), 3.52–3.61 (2H,s), 3.72–3.81(4H, d), 3.91–4.01 (4H, s), 4.2 (3H,m), 4.71–4.81 (4H,m), 5.23–5-41(2H,m), 5.61–5.81(1H, m), 6.25–6.4(2H,m), 6.45–6.61(4H,m), 6.8 (2H,s). ESI MS: m/z found, 1340.84828 (M+H), calcd 1339.88788.

### Synthesis of nimotuzumab ADCs

Nimotuzumab or control human IgG (maltose binding protein (MBP IgG) was conjugated with DM1-PEG_6_-NHS. A 20 mg/mL stock solution (in DMSO) of DM1-PEG_6_-NHS was prepared. The conjugation reactions were optimized for pH, buffers and reaction time. In order to prepare ADCs with low (DAR 3–4) and high (DAR 7–8), a 5–50 mole excess equivalent of drug linker DM1-PEG_6_-NHS was used to optimize the conjugation of the antibodies to obtain a low (nimotuzumab-PEG_6_-DM1-Low) or high (nimotuzumab-PEG_6_-DM1-High) DAR. Nimotuzumab (5 mg/mL in PBS) was buffer exchanged in centrifugal filters (Amicon Ultra-4 Centrifugal Filter 10K NMCO, EMD Millipore, Burlington, MA, USA) and allowed to react with the DM1-PEG_6_-NHS for 3–20 h at ambient temperature. In all reactions the amount of DMSO was kept at <3%. The excess unconjugated drug linker was removed from reaction mixture using centrifugal filters and PBS as a storage buffer. All the conjugates were passed through 0.2 μM membrane filters and aliquoted into 20 μL vials. The vials were stored at −80° C until further use. The DAR was then determined by UV spectrophotometry [39] and Bioanalyzer (Mississauga, ON, USA).

### Quality control of ADCs

Quality control was done on all batches of nimotuzumab ADCs and control immunoconjugates. Size exclusion HPLC (SEC-HPLC) using Waters 2796 Bioseparations Module, Waters 2487 Dual λ Absorbance Detector, XBridge^®^ BEH 200A SEC 3.5 μm 7.8 × 300 mm column (Waters Corporation, Milford, MA, USA) was used to determine the integrity of ADCs and free drug. The UV-Detector was set at 254 and 280 nm with PBS as the solvent and a flow rate of 0.6 mL/min. The size and purity of ADCs was characterized by electronic electrophoresis (bioanalyzer). The analysis of molecular weight and purity of all the conjugated samples were performed on an Agilent 2100 Bioanalyzer system using Agilent High Sensitivity Protein 250 Kit (cat # 5067-1575) according the manufacturer's protocol.

### Binding kinetics of ADCs

Binding kinetics between antibodies and target proteins were measured using ForteBio Octet RED384 (PALL Corporation, New York, NY, USA). Antibodies were immobilized on anti-human FAB-CH1 sensors (18-5104, Forte Bio) according to manufacturer's instructions. After immobilization, antibodies were exposed to 500 nM, 166 nM, and 55 nM concentrations of the 9x-His tagged monomeric hEGFR, Leu 25 - Ser 645 (1001-H08H, Sino Biological) proteins. At the same time, empty sensors were exposed to the same concentrations of the target protein to be used for subtraction of non-specific binding. All reactions were performed at 30 °C in 1× kinetics buffer (18-5032, Forte Bio). The equilibrium dissociation constant (K_D_) was obtained using a 1 to 1 model with global fitting. Data analysis and curve fitting was performed using data analysis software 7.1.0.33 (Forte Bio).

### Flow cytometry

*In vitro* binding to EGFR positive cells was performed for all immunoconjugates and compared with unconjugated nimotuzumab or control anti-MBP antibody. 10^5^ cells (DLD-1 or HT-29) were collected and washed with PBS containing 2% FBS and seeded on either 96 well plate or FACS tubes. Nimotuzumab and ADCs were titrated at a minimum of a 10-fold excess onto cells (single-point 10 μM and eight-point assays starting from 0.1 μM). Similarly, anti-MBP antibody and its ADCs were prepared as control immunoconjugates. Cells treated with all compounds were incubated for 30 minutes at room temperature followed by 15 minutes on ice. Cells were washed and resuspended in a 1:50 dilution of FITC labeled Goat F(ab’)2 fragment anti-human IgG (H + L) antibody (Beckman Coulter, IM0839) and incubated for 30 minutes on ice in the dark. Cells were washed and suspended in 2% FBS-PBS. Data was acquired using a MACSquant VYB (Miltenyl biotech) and the results were analysed using FlowJoV10. The binding constant for each conjugate was generated using GraphPad prism 6.

### *In vitro* cytotoxicity and apoptosis

The cytotoxicity of nimotuzumab and ADC immunoconjugates was studied in DLD-1, MBA-MB-468 and HT-29 cells using IncuCyte S3 Live cell imaging system (Essen BioScience, Ann Arbor, MI, USA). Briefly, 20,000 cells were seeded 24 h prior to treatment in a 96 well clear bottom corning pre-coated with Poly-D-lysine plates. The next day, the media was removed and washed with PBS. Cells were then incubated with IncuCyte^®^ Cytotox Red reagent diluted in complete media (1×, Essen Bioscience Cat #4632) for 3 h before treatment. Cells were treated with different concentrations (0.2–500 nM) of nimotuzumab or nimotuzumab-PEG_6_-DM1-High or nimotuzumab-PEG_6_-DM1-Low and incubated at 37° C for 30 min prior to scanning. Live cell images were captured every 2 h for 72 hours using a 10× objective lens using phase contrast and fluorescence channel. During each scanning 5 images were acquired until the end of the experiment. All cell images were processed and analysed using IncuCyte S3 software. Relative fluorescent values were generated and IC_50_ values for individual compounds were calculated using GraphPad prism 6.

### Normal tissue toxicity

Delayed (Day-14) toxicity studies were carried out using six-week old Balb-C male and female mice (*n* = 5 males + 5 females) (Charles Rivers, Senneville, QC, Canada). Mice were monitored for weight and behaviour changes. Nimotuzumab, nimotuzumab-PEG_6_-DM1-Low and nimotuzumab-PEG_6_-DM1-High were formulated in PBS; PBS was used as a control. Groups of mice (5 males + 5 females) were injected with 15 mg/kg, 25 mg/kg or 50 mg/kg of ADC, antibody or PBS and sacrificed on Day14. All animals in this study were observed regularly for signs of mortality, morbidity, injury, and food and water intake. Individual body weights were recorded during the quarantine period (every other day) and experimental period. Blood was collected via cardiac puncture for cell blood counts (CBC) and clinical chemistry. Organs of interest (kidneys, spleen, liver, bone, heart, lungs, brain, and testes/uterus) were harvested and stored in 10% formaldehyde solution. They were further processed and examined for histopathological analyses.

### Efficacy of ADCs in mouse xenograft

All animals used in imaging experiments were cared for and maintained under the supervision and guidelines of the University of Saskatchewan Animal Care Committee (protocol # 20160005). Female athymic CD-1 nude mice were obtained from Charles River Canada (Senneville, QC) at 4 weeks of age and housed in a 12 h light, 12 h dark cycle, temperature and humidity controlled vivarium. Animals had ad libitum access to mouse diet (Lab Diet, St. Louis, Missouri, USA) and water. Generation of near infrared labeled DLD-1-IRFP-702 cells used for *in vivo* efficacy studies is described in Supplementary Materials. After one week of acclimatization, mice were subcutaneously injected with a suspension of 10^7^ DLD-1-IRFP-702 cells in 100 μL of a 1:1 mixture of serum-free RPMI-1640 medium (HyClone Laboratories, Logan, Utah) and matrigel matrix basement membrane (Discovery Laboware, Inc. Bedford, MA, USA) in the right hind limb of each mouse. Tumor growth was followed by measuring the greatest length and the greatest width from each tumor using an external caliper. Then tumor volume was calculated using the formula: tumor volume = length × width^2^ × 0.5. When xenografts average size measured 150 mm^3^ in volume, mice were randomized into 5–6 mice per group and each mouse was injected intravenously with normal saline or 15 mg/kg body weight of either nimotuzumab, nimotuzumab-PEG_6_-DM1-Low or nimotuzumab-PEG_6_-DM1-High via a tail vein on day 0, 8, and 16. The DLD-1-IRFP-702 tumor bearing mice were anesthetized with 2.5% isoflurane and imaged at different time points using the Pearl Impulse Imager (LI-COR) near infrared scanner. The excitation/emission settings were 685/720 nm. The fluorescence signal was overlaid with the white light image captured by a CCD camera of the imager.

### Statistical analysis

All data was expressed as the mean ± SD or SEM of at least 3 independent experiments. Statistical significance between groups was assessed using a two-tailed Student's *t-*test or analysis of variance (ANOVA) with Bonferoni post hoc test. All graphs were prepared and analysed using GraphPad Prism (version 5; GraphPad, La Jolla, CA, USA).

## SUPPLEMENTARY MATERIALS FIGURES AND TABLES



## Abbreviations

SPECT: single photon emission computed tomography
EGFR: epidermal growth factor receptor I
DOTA: 1,4,7,10-Tetraazacyclododecane-1,4,7,10-tetraacetic acid
%IA: percent injected activity
%IA/g: percent injected activity per gram
NIR: near infrared imaging
SEC: size exclusion column
mCRC: metastatic colorectal cancer
KRAS: Kirsten RAS oncogene
