# Supplementary material for: Therapeutic potential of nimotuzumab PEGylated-maytansine antibody drug conjugates against EGFR positive xenograft
Source: Oncotarget. 2019 Feb 1;10(10):1031–44. doi: 10.18632/oncotarget.26613 (PMC6383682; doi:10.18632/oncotarget.26613)
Supplement: Supplementary file 1 [file oncotarget-10-1031-s001.pdf]

Therapeutic potential of nimotuzumab PEGylated-maytansine antibody drug conjugates against EGFR positive xenograft

SUPPLEMENTARY MATERIALS

Development of DLD-1-iRFP-702 near infrared labeled cell line

Cell-lines expressing iRFP-702 were constructed by transduction with a lenti-viral plasmid containing iRFP702 under the human PGK promoter as follows. The GFP-IRES-LUC cassette in pHAGE-PGK-GFP-IRES-LUC-W was replaced with iRFP702 PCR amplified from iRFP-702-N1 (Addgene#45456) with primers. (5′-CTC CGG GCC TTT CGA CCT GCG GCC GCC ATG GCG CGT AAG GTC GAT CTC A-3′) and (5′-ATC CAG AGG TTG ATT AGG ATC TAT CGA TTT AGC GTT GGT GGT GGG CGG C-3′). pHAGE-PGK-GFP-IRES-LUC-W was digested with Not1 and Cla1. The iRFP-702 encoding DNA was cloned into *Not1* and *Cla1* digested pHAGE-PGK plasmid using Gibson Assembly to produce pHAGE-PGK-iRFP702.

Virus was produced in HEK293 cells by PEI transfection of PHAGE-PGK-iRFP702. Five million HEK293 cells were seeded in a 6-well plate and grown overnight at 37° C at 5% CO_2. The next day 2 µg of total DNA was transfected into HEK293 cells as previously described [1]. The virus was collected 72 hours later and filtered through a 0.22 µ filter. DLD-1 (colorectal cancer) cell line was transduced at a multiplicity of infection (MOI) of approximately 0.1 to ensure the majority of cells had a single infection [1]. To obtain a stable population for DLD-1-iRFP-702 cells were then sorted for iRFP positive cells, expanded and re-sorted. Cells were then monitored by flow cytometry. At 11 passages no change in the percent of positive cells was observed.

Quantification of tumor response to treatment using near infrared imaging (NIR)

To obtain more accurate tumor volumes NIR images obtained using Pearl Imager were analyzed. Images were analyzed using R: The R project for statistical computing [2], using packages RBioFormats [3] to import images, EBImage [4] to segment images, data table to manage data, and ggplot2 [5] and plotly [6] for plotting images. Images were despeckled for instrument noise using the medianFilter function with parameters size = 3. Noise was determined by inspecting the contralateral region of mice and determining the maximum signal in an area with no tumour defined for this study as 0.08. We then identified objects using the bwlabelfunction with parameters threshold = noise*signal:noise, using a robust sn ratio of 10-fold. All images were processed identically. Information was extracted including area, perimeter, mean radius, error of radius, min radius and max radius about the tumor. No more than one object was detected in all images indicating no artifacts were detected using these settings.

REFERENCES

1. Bernhard W, Barreto K, Raithatha S, Sadowski I. An upstream YY1 binding site on the HIV-1 LTR contributes to latent infection. PLoS One. 2013; 8:e77052.
2. R Core Team. R: A language and environment for statistical computing. R Foundation for Statistical Computing. 2017. Vienna, Austria. https://www.R-project.org/.
3. Andrzej Oleś. RBioFormats: R interface to Bio-Formats. R package version 0.0.34. https://rdrr.io/github/aoles/RBioFormats/man/RBioFormats.html.
4. Pau G, Fuchs F, Sklyar O, Boutros M, Huber W. EBImage--an R package for image processing with applications to cellular phenotypes. Bioinformatics. 2010; 26:979–981.
5. Matt Dowle and Arun Srinivasan. data.table: Extension of `data.frame`. R package version 1.10.4. 2017. https://CRAN.R-project.org/package=data.table.
6. H. Wickham. ggplot2: Elegant Graphics for Data Analysis. New York, USA: Springer-Verlag; 2009.

Supplementary Table 1A: Cell blood counts (CBC) of Female and male mice after tail vein injection of Nimotuzumab-PEG$_6$-DM1-Low 15, 25 and 50 mg/kg (*n* = 5)

	Nimotuzumab-PEG$_6$-DM1-Low					
**Parameter**	**Females**			**Males**		
	**15 mg/kg**	**25 mg/kg**	**50 mg/kg**	**15 mg/kg**	**25 mg/kg**	**50 mg/kg**
WBC × 10^9/L	1.4 ± 0.2	2.6 ± 0.6†	3.2 ± 0.5†	1.5 ± 0.2	2.3 ±0.4*	2.9 ± 0.5*
LY %	86.9 ± 5.4	75.2 ± 1.5†	73.0 ± 1.9†	86.2 ± 2.3	77.6 ± 2.4†	67.9 ± 2.3†
MO %	10.2 ± 4.5	15.7 ± 1.7*	16.0 ± 2.1*	11.7 ± 2.6	16.1 ±2.3*	19.4 ± 2.4†
GR %	2.7 ± 2.2	8.9 ± 2.3*	8.1 ± 0.2*	2.0 ± 0.4	6.3 ± 2.6*	11.4 ± 5.1†
LY# × 10^9/L	1.2 ± 0.2	1.6 ± 1.0	2.0 ± 0.7	1.3 ± 0.2	1.5 ± 0.5	1.1 ± 0.5
MO# × 10^9/L	0.2 ± 0.2	0.3 ± 0.2	0.3 ± 0.2	0.2 ± 0.1	0.3 ± 0.1	0.3 ± 0.1
GR# × 10^9/L	0.1 ± 0.01	0.2 ± 0.1	0.2 ± 0.0*	0.1 ± 0.0	0.2 ± 0.5	0.4 ± 0.1
RBC × 10^{12}/L	4.21 ± 0.1	4.1 ± 0.6	4.0 ± 0.1*	4.3 ± 0.1	4.1 ± 2.1	3.8 ± 0.1†
Hgb g/L	71.8 ± 2.7	70.6 ± 9.4	66.7 ± 2.0*	70.2 ±2.1	79.8 ± 3.6	63.4 ± 2.1†
Hct L/L	0.21 ± 0.1	0.2 ± 0.01*	0.2 ± 0.0*	0.2 ± 0.1	0.2 ± 0.0	0.2 ± 0.1
MCV fL	50.1 ± 0.4	52.3 ± 0.8	45.7 ± 0.1*	50.6 ± 0.4	50.2 ±0.5	49.6 ± 0.7
MCH pg	16.5 ± 0.3	16.1 ± 0.8	16.7 ± 0.1*	15.9 ± 0.9	16.1 ± 0.2	16.1 ± 0.4
MCHC g/L	324 ± 6.7	315 ± 2.3	308 ± 1.1†	321 ± 8.2	317 ± 1.7†	312 ± 4.2†
RDW %	16.4 ± 0.5	18.6 ± 0.1*	18.9 ± 0.1*	17.1 ± 0.5	18.1 ± 0.5*	23.7 ± 3.8†
Plt × 10^9/L	413 ± 10	401 ± 7	339 ± 5†	460 ± 28	429 ± 29	377 ± 15†
MPV fL	5.6 ± 0.3	5.9 ± 0.5	5.6 ± 0.6	5.7 ± 0.4	5.9 ± 0.8	6.0 ± 1.4

All the results were compared with control group treated with PBS. The statistical difference is indicated by $^*p < 0.05$, $^†p < 0.01$. These values were compared with those of PBS treated mice (omitted from table).

Supplementary Table 1B: Cell blood counts (CBC) data from the mice receiving 15 and 25 mg/kg of Nimotuzumab-PEG$_6$-DM1-High (*n* = 5)

	Nimotuzumab-PEG$_6$-DM1-High			
	**Female**		**Males**	
**Parameter**	**15 mg/kg**	**25 mg/kg**	**15 mg/kg**	**25 mg/kg**
WBC × 10^9/L	1.52 ± 0.1	2.2 ± 0.2†	1.5 ± 0.22	2.4 ± 0.2†
LY %	88.2 ± 3.2	86.2 ± 5.5	83.2 ± 2.8*	89.5 ± 4.1
MO %	9.07 ± 2.9	10.0 ± 3.6	9.2 ± 3.9	15.4 ± 4.3*
GR %	1.7 ± 0.6	1.7 ± 0.8	1.2 ± 0.3	3.1 ± 0.7*
LY# × 10^9/L	1.6 ± 0.4	1.4 ± 0.2	1.7 ± 0.1*	1.3 ± 0.2
MO#× 10^9/L	0.2 ± 0.13	0.2 ± 0.05	0.2 ± 0.1	0.24 ± 0.1
GR# × 10^9/L	0.1 ± 0.01	0.1 ± 0.01	0.1 ± 0.01	0.1 ± 0.01
RBC × 10^{12}/L	4.6 ± 0.75	4.0 ± 0.2*	4.4 ± 0.12	4.0 ± 0.18†
Hgb g/L	75.5 ± 1.2	69.8 ± 3.2	70.8 ± 0.9	64.6 ± 2.7*
Hct L/L	0.2 ± 0.03	0.3 ± 0.03	0.2 ± 0.01	0.21 ± 0.07
MCV fL	50.5 ± 0.4	51.4 ± 0.8	50.0 ± 1.1	48.7 ± 1.6
MCH pg	16.5 ± 0.13	16.7 ± 0.15	16.5 ± 0.1	16.5 ± 0.1
MCHC g/L	331 ± 11	345 ± 18	324 ± 3.8	328 ± 2.1
RDW %	17.2 ± 0.1*	18.01 ± 0.3†	18.0 ± 1.1	18.1 ± 1.4*
Plt × 10^9/L	509 ± 10	525 ± 74*	499 ± 36	558 ± 59*
MPV fL	6.2 ± 0.4	6.2 ± 0.6	5.3 ± 0.3	5.9 ± 0.5

The statistical difference was indicated by $^*p < 0.05$, and $^†p < 0.01$. These values were compared with those of PBS treated mice (omitted from table).

Supplementary Table 2A: Serum biochemistry from the mice injected with 15, 25 and 50 mg/kg of Nimotuzumab-PEG$_6$-DM1-Low (n = 5)

	Nimotuzumab-PEG$_6$-DM1-low					
	Female			Male		
**Parameters**	**15 mg/kg**	**25 mg/kg**	**50mg/kg**	**15 mg/kg**	**25 mg/kg**	**50 mg/kg**
Sodium mmol/L	147 ± 0.7	149 ±1.7	147 ± 1.8	148 ± 0.9	146 ± 2.0	147 ± 1.1*
Potassium mmol/L	5.4 ± 0.7	5.4 ± 2.1	5.0 ± 0.3	5.7 ± 0.7	5.3 ± 0.5	4.9 ± 0.3*
Na:K Ratio	28.5 ± 1.3	30.7 ± 1.9	30 ±2.5	26 ± 3.6	27.3 ± 0.5	30 ± 1.1*
Chloride mmol/L	107 ± 0.7	109 ± 1.5	105 ± 1.5	106 ± 1	105 ± 1.1	102 ± 1.5
Bicarbonate mmol/L	14 ± 1.8	14 ± 2.3	16 ± 1.2*	15.7 ± 0.5	17 ± 1	18 ± 1.2*
Anion Gap mmol/L	30 ± 3.5	30 ± 4.4	29.5 ± 1	31.7 ± 1.5	29 ± 1.7	29.5 ± 0.1*
Calcium mmol/L	2.4 ±0.05	2.1 ± 0.4	2.6 ± 0.0*	2.4 ± 0.1	2.3 ± 0.07	2.5 ± 0.04
Phosphorus mmol/L	2.4 ± 0.5	2.5 ± 0.1	2.1 ± 0.1	2.1 ± 0.2	1.9 ± 0.3*	1.28 ± 0.1**
Magnesium mmol/L	0.9 ± 0.0	0.7 ± 0.3	0.8 ± 0.0	0.8 ± 0.1	0.7 ± 0.1	0.7 ± 0.04*
Urea mmol/L	9 ± 0.5	7.6 ± 0.3	8.1 ± 0.8	8.5 ± 0.7	7.5 ± 0.3	8.0 ± 0.4
Creatinine µmol/L	12 ± 1.8	18 ± 1*	20 ± 0.1**	13.2 ± 1.2	18 ± 1.0*	21 ± 1.6**
Amylase U/L	2644 ± 340	3034 ± 303	3594 ± 0.4*	2979 ± 592	3477 ± 634	3294 ± 440*
Lipase U/L	25 ± 2.8	23 ± 1.5	31 ± 0.1*	27.5 ± 1.2	29.1 ± 2.0	30.2 ± 1.2*
Glucose mmol/L	11 ± 1.2	12 ± 1.6	18 ± 0.1*	12.6 ± 0.6	13. 9 ± 0.2	15.8 ± 0.1**
Cholesterol mmol/L	2.4 ±0.1	2.5 ± 0.1	3.3825	3.4 ± 0.4	3.2 ± 0.1	3.3 ± 0.1
Total Bilirubin µmol/L	0.7 ± 0.2	0.8 ± 0.1	0.1 ± 0.1***	1.3 ± 0.2	0.5 ± 0.4	0.1 ± 0.2***
Direct Bilirubin µmol/L	0.3 ± 0.14	0.4 ± 0.1	0.1 ± 0.1**	0.6 ± 0.1	0.3 ± 0.2	0.1 ± 0.1**
Indirect Bilirubin µmol/L	0.5 ± 0.1	0.45 ± 0.1	0.01 ± 0.1*	0.7 ± 0.2	0.2 ± 0.2	0.1 ± 0.0*
Alk phos U/L	119 ± 1.8	149 ± 1.8*	159 ± 1*	104 ± 9.6	131 ± 3.6	148 ± 6.2***
ALT U/L	39 ±2.8	51 ±2*	72 ± 1**	59 ± 1.9	74.6 ± 4.2*	92 ± 3.5**
GLDH U/L	12 ± 2.9	14 ± 1	18 ± 1*	14.6 ± 4.1	10 ± 2.6	14.7 ± 1.7
CK U/L	1909 ± 314	1746 ± 163	1109 ± 98*	3918 ± 267	1111 ± 979*	1109 ± 119*
Total Protein g/L	47 ± 4.2	46 ± 1	51 ± 0.9	49 ± 0.8	47.6 ± 0.5*	41.3 ± 0.9**
Albumin g/L	31 ± 2	28 ± 1	29 ± 1	29.5 ±1.1	28.3 ± 1.1	29.2 ± 0.5
Globulin g/L	15 ± 2	18 ± 1	22 ± 1	20 ± 0.8	19.3 ± 0.5*	18.1 ± 0.4*

All the mice were monitored for 14 days and blood was collected by cardiac puncture. The statistical difference was indicated by $^{*}p < 0.05$, $^{**}p < 0.01$ and $^{***}p < 0.001$. These values were compared with those of PBS treated mice (omitted from table).

Supplementary Table 2B: serum biochemistry results from the mice injected with 15 and 25 mg/kg of Nimotuzumab-PEG$_6$-DM1-High (n = 5)

	Nimotuzumab-PEG$_6$-DM1-High			
**Parameters**	**15 mg/kg**	**25 mg/kg**	**15 mg/kg**	**25 mg/kg**
Sodium mmol/L	152 ± 0.5*	153 ± 0.1*	153 ± 0.3*	153 ± 0.1*
Potassium mmol/L	4.2 ± 0.3	4.6 ± 0.4	4.85 ± 0.1*	5.0 ± 0.1*
Na:K Ratio	36.3 ± 2.8	35.8 ± 3.4	31.8 ± 3.2*	31 ± 2.1*
Chloride mmol/L	110 ± 1.0	108 ± 0.7	111 ± 0.9	109 ± 1
Bicarbonate mmol/L	14 ± 0.5	15 ± 0.5	15 ± 0.8	17.6 ± 0.9
Anion Gap mmol/L	32 ± 2.0	31 ± 2.6	31.5 ± 1.1	31.3 ± 0.4
Calcium mmol/L	2.4 ± 0.0	2.4 ± 0.1	2.4 ± 0.2	2.4 ± 0.1
Phosphorus mmol/L	2.5 ± 0.23	2.6 ± 0.4	2.2 ± 0.4	2.4 ± 0.2
Magnesium mmol/L	1.0 ± 0.1	1.6 ± 0.1*	0.8 ± 0.2	0.9 ± 0.1
Urea mmol/L	7.5 ± 1.0	8.85	8.9 ± 1.1	8.2 ± 0.9
Creatinine µmol/L	15.3 ± 0.3	21.7 ± 0.4**	14.2 ± 0.8	18.1 ± 0.2
Amylase U/L	2834 ± 250	3154 ± 111*	3511 ± 373	2865 ± 212
Lipase U/L	24 ± 1.5	22.5 ± 0.5*	24.1 ± 1.4	21.1 ± 0.5*
Glucose mmol/L	10.7 ± 0.2	10.15	12.5 ± 0.8	15.1 ± 0.1**
Cholesterol mmol/L	3.0 ± 0.2	2.8 ± 0.2*	3.4 ± 0.2	3.4 ± 0.1
Total Bilirubin µmol/L	0.9 ± 0.5	0.1 ± 0.1***	0.85 ± 0.2	0.1 ± 0.1***
Direct Bilirubin µmol/L	0.6 ± 0.1	0.1 ± 0.1**	0.6 ± 0.1	0.1 ± 0.2**
Indirect Bilirubin µmol/L	0.5 ± 0.1	0.2 ± 0.1*	0.3 ± 0.3	0.1 ± 0.1*
Alk phos U/L	135 ± 7.9*	151 ± 10**	114 ± 20	116 ± 3*
ALT U/L	35.3 ± 1.6	70.5 ± 3.1**	54 ± 9.8	73 ± 4.1**
GLDH U/L	9 ± 0.5	7.5 ± 0.5*	14.3 ± 6.2	8.6 ± 0.5
CK U/L	1972 ± 252	1078 ± 468*	2108 ± 497*	1101 ± 102**
Total Protein g/L	49 ± 0.5	48 ± 1.8	49 ± 0.9	48 ± 1.4
Albumin g/L	31 ± 0.5	29 ± 0.1*	25.7 ± 2.8	21.7 ± 1.0*
Globulin g/L	18 ± 0.5	18 ± 1.2	20 ± 1.5	23 ± 2.1

The statistical difference was indicated by $^*p < 0.05$, $^{**}p < 0.01$ and $^{***}p < 0.001$. These values were compared with those of PBS treated mice (omitted from table).

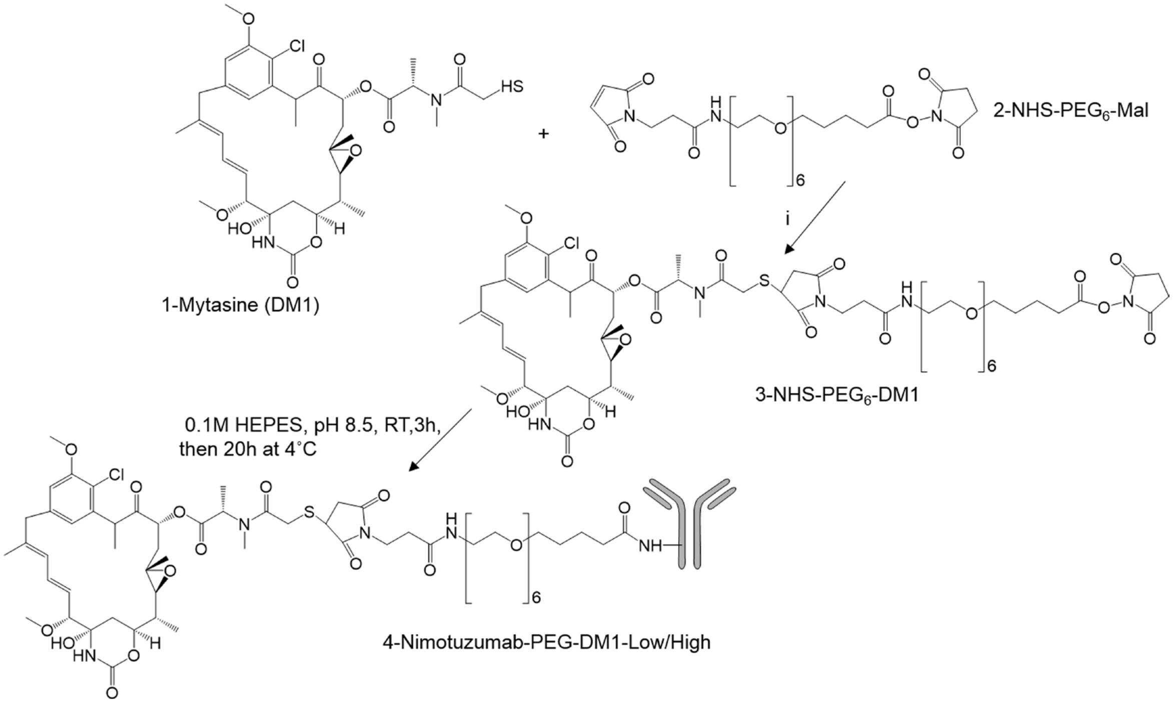

Supplementary Scheme 1: Synthetic scheme of nimotuzumab drug conjugation. Maytansine (DM1) was reacted with bifunctional linker NHS-PEG$_6$-Mal (2) in 50 mM PBS/THF for 6 h at room temperature to generate the NHS-PEG$_6$-DM1. NHS-PEG$_6$-DM1 was analyzed by mass spectrometry and NMR. Different fold excess of NHS-PEG$_6$-DM1 was then reacted with nimotuzumab in 0.1 M HEPES pH 8.5 at room temperature for 3 h followed by 4° C for 20 h to yield nimotuzumab-PEG$_6$-DM1-Low (3–4 drugs per antibody) or nimotuzumab-PEG$_6$-DM1-High (7–8 drugs per antibody).

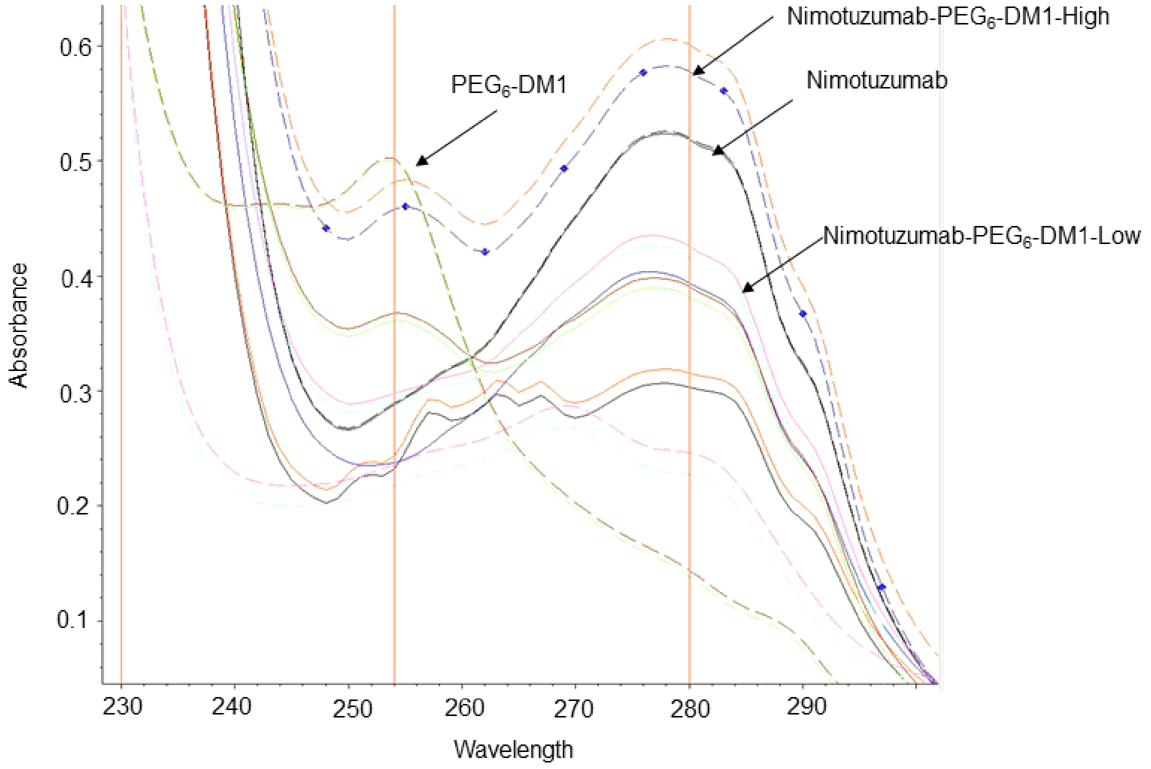

Supplementary Figure 1: Determination of Antibody-drug-ratio (UV method). Distinct UV spectra of drug, antibody and antibody drug conjugates are shown. Drug to antibody ratio was determined using UV method. Antibody and drug each has absorbance maxima at different wavelengths i.e. 280 and 254 nm respectively. Simultaneous equations were generated for both drug and antibody using the Beer–Lambert law. By solving the simultaneous equations, the number of drug per antibody was determined.

Equation 1: Antibody- Absorption at 280 nm

$$A_{280} = (\varepsilon_{drug}^{280} C_{drug} + \varepsilon_{mAb}^{280} C_{mAb}) l$$

Equation 2: Antibody- Absorption at 254 nm

$$A_{254} = (\varepsilon_{drug}^{254} C_{drug} + \varepsilon_{mAb}^{254} C_{mAb}) l$$

The average drug to antibody ratio can be determined by dividing the Cdrug by Cmab and is expressed moles of drug to moles of antibody.

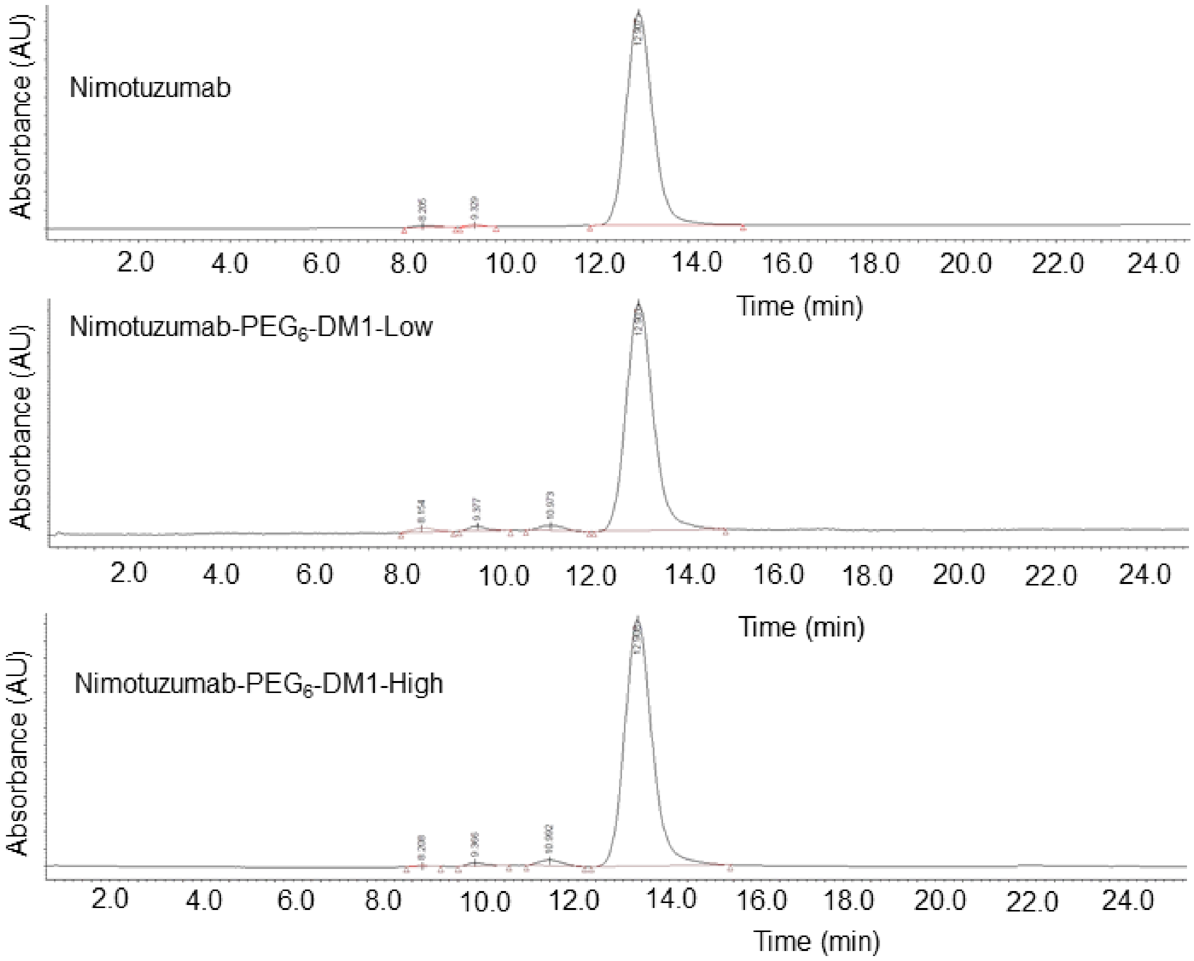

Supplementary Figure 2: Size exclusion HPLC chromatograms of nimotuzumab, nimotuzumab-PEG$_6$-DM1-Low and nimotuzumab-PEG$_6$-DM1-High.

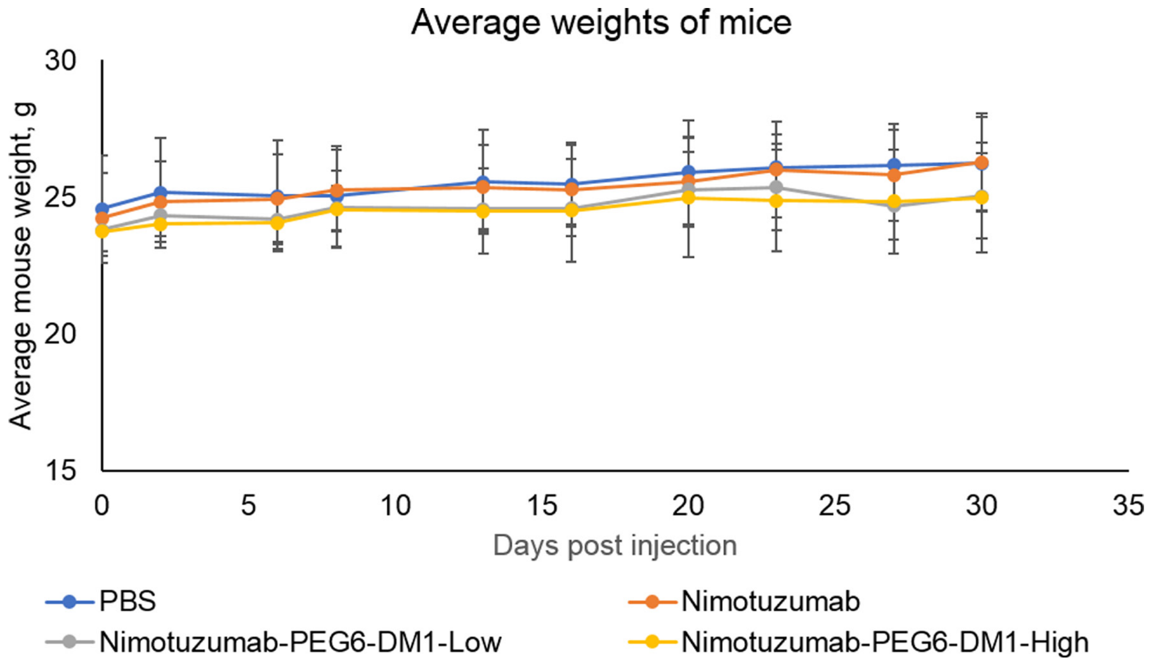

Supplementary Figure 3: Body weight changes of mice treated with 15 mg/kg nimotuzumab, nimotuzumab-PEG$_6$-DM1-Low and nimotuzumab-PEG$_6$-DM1-High.

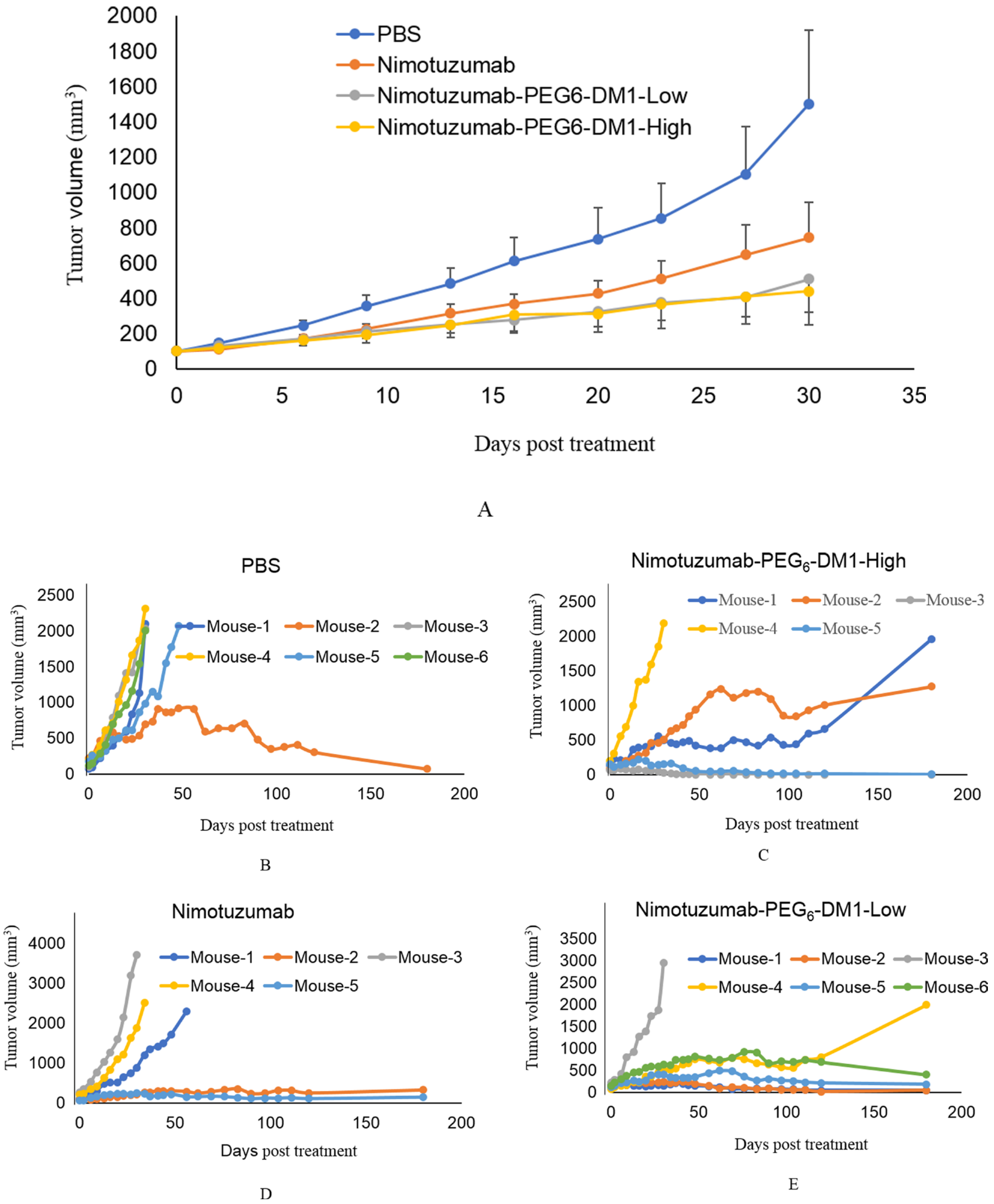

Supplementary Figure 4: Tumor growth cures of DLD-1-iRFP-702 xenograft measured using a digital caliper after treatment with PBS, nimotuzumab, nimotuzumab-PEG$_6$-DM1-Low and nimotuzumab-PEG$_6$-DM1-High. (**A**) Average tumor growth of the different groups – For all groups, these curves represent average tumor volume when all mice were alive (no mouse had reached treatment endpoint). (**B**) Tumor growth of each mouse treated with PBS. Curves plotted with values from start of treatment till end of study (180 days). (**C**) Tumor growth of each mouse treated with nimotuzumab-PEG$_6$-DM1-High. Curves ploted with values from start of treatment till end of study (180 days). (**D**) Tumor growth of each mouse treated with nimotuzumab. Curves plotted with values from start of treatment till end of study (180 days). (**E**) Tumor growth of each mouse treated with nimotuzumab-PEG$_6$-DM1-Low. Curves plotted with values from start of treatment till end of study (180 days).
